# Trends in and predictors of pregnancy termination among 15–24 year-old women in Nigeria: a multi-level analysis of demographic and health surveys 2003–2018

**DOI:** 10.1186/s12884-020-03164-8

**Published:** 2020-09-22

**Authors:** Franklin I. Onukwugha, Monica A. Magadi, Ahmed M. Sarki, Lesley Smith

**Affiliations:** 1 Institute for Clinical and Applied Health Research, Faculty of Health Sciences University of Hull, Hull, UK; 2 Department of Criminology and Sociology, Faculty of Arts, Cultures and Education, University of Hull, Hull, UK; 3 School of Nursing and Midwifery, Aga Khan University (East Africa campus), Kampala, Uganda

**Keywords:** Maternal health, Adolescence, Health inequalities, Sustainable development goals, Prevalence, Low middle income countries

## Abstract

**Background:**

Three-quarters of pregnancy terminations in Africa are carried out in unsafe conditions. Unsafe abortion is the leading cause of maternal mortality among 15–24 year-old women in Sub-Saharan Africa. Greater understanding of the wider determinants of pregnancy termination in 15–24 year-olds could inform the design and development of interventions to mitigate the harm. Previous research has described the trends in and factors associated with termination of pregnancy for women of reproductive age in Nigeria. However, the wider determinants of pregnancy termination have not been ascertained, and data for all women have been aggregated which may obscure differences by age groups. Therefore, we examined the trends in and individual and contextual-level predictors of pregnancy termination among 15–24 year-old women in Nigeria.

**Methods:**

We analysed data from the 2003, 2008, 2013 and 2018 Nigerian Demographic and Health Surveys (NDHS) comprising 45,793 women aged 15–24 years. Trends in pregnancy termination across the four survey datasets were examined using bivariate analysis. Individual and contextual predictors of pregnancy termination were analysed using a three-level binary logistic regression analysis and are reported as adjusted odds ratios (aOR) with 95% confidence intervals (CI).

**Results:**

Trends in pregnancy termination declined from 5.8% in 2003 to 4.2% in 2013 then reversed to 4.9% in 2018. The declining trend was greater for 15–24 year-old women with higher socioeconomic status. Around 17% of the total variation in pregnancy termination was attributable to community factors, and 7% to state-level factors. Of all contextual variables considered, only contraceptive prevalence (proxy for reproductive health service access by young women) at community level was significant. Living in communities with higher contraceptive prevalence increased odds of termination compared with communities with lower contraceptive prevalence (aOR = 4.2; 95% CI 2.7–6.6). At the individual-level, sexual activity before age 15 increased odds of termination (aOR = 2.3; 95% CI 1.9–2.8) compared with women who initiated sexual activity at age 18 years or older, and married women had increased odds compared with never married women (aOR = 3.0; 95% CI 2.5–3.7).

**Conclusion:**

Our findings highlight the importance of disaggregating data for women across the reproductive lifecourse, and indicates where tailored interventions could be targeted to address factors associated with pregnancy termination among young women in Nigeria.

## Background

Pregnancy termination or induced abortion, defined as a pregnancy that is terminated by choice through intervention, is a major public health issue in Africa [1]. Abortion services in Africa are restricted with only 4 out of 54 countries having relatively liberal abortion laws [1]. Between 2010 and 2014, of the 6.86 million abortions carried out each year in Africa, 75.6% (95% confidence interval (CI) 66.4 to 81.4%) of these were classified as unsafe (using untrained practitioners and/or non-recommended methods) [2]. Unsafe abortion has serious adverse health consequences such as infections and fatality [2]. Young women aged 15–24 years account for 57% of abortions in Sub-Saharan Africa (SSA) [3], with unsafe abortion being the leading cause of maternal mortality in this age group [4–7].

In Nigeria, the situation is further exacerbated by socio-cultural and religious beliefs whereby termination of pregnancy is viewed as murder and carries harsh penalties for providers [8, 9]. These factors contribute to women seeking clandestine abortion services with private providers who are mostly untrained, carry out procedures in unsafe environments using dangerous invasive methods [10, 11]. Notwithstanding, the punitive abortion laws in Nigeria, in 2012 the incidence of induced abortion was estimated as 33 per 1000 women [12]. More recently a pooled analysis of the Nigerian Demographic and Health Survey data (NDHS) for 2003–2013 reported the percent of married women who had ever terminated a pregnancy as 3.8% (95% CI 3.3 to 4.2%) amongst 15–19-year olds, and 11.7% (95% CI 11.0 to 12.5%) for 20–24-year olds [13].

While much research has been published reporting on young people’s knowledge, attitudes, practices and prevalence of pregnancy termination in SSA especially in Nigeria [3, 9, 12, 14], fewer studies have investigated factors influencing termination. The prevalence of pregnancy termination is higher in women of older age [8, 15, 16]; young age of sexual debut [17]; employed women [13], unintended pregnancy [8, 16], forced or transactional sex [18], physical or sexual intimate partner violence [19, 20], higher number of living children [18, 21], and higher wealth index [13, 22]. Being married [8, 13, 22, 23], having a secondary education [15, 22, 24], modern contraceptive use [23] and residing in rural areas have been associated with lower prevalence of induced abortion [13, 15].

Socio-cultural factors such as religion, mass media, and ethnicity have also been found to influence termination of pregnancy among adolescents and young people [8]. At the community and societal levels, place of residence, region of residence, community (mean education, attitudes justifying wife-beating, median age of first marriage, contraceptive use) and facility-level factors (policy, limited availability of health facilities providing abortion services and lack of patient-centredness of health services) have been noted to significantly predict termination of pregnancy [20, 23, 25].

Although the evidence above sheds light on some factors that are associated with pregnancy termination in women overall, they fall short of ascertaining the determinants specifically for 15–24 year-old women in Nigeria. The studies that involved young people were of adolescents in school [16, 17] and sample sizes were small and not representative of the general population, limiting inferences that can be drawn from the findings. Moreover, the studies largely focused on individual-level socio-economic and demographic factors with limited attention on the contextual community and state-level factors that influence pregnancy termination. Ignoring these factors especially in Nigeria with wide variation in socio-economic/cultural and regional characteristics hampers development of interventions to mitigate early pregnancy among 15–24 year-old women [26]. Furthermore, empirical studies outside of SSA have shown that contextual level factors such as cultural norms, inadequate quality or lack of sexual health education in schools, and lack of youth-friendly services may influence pregnancy termination among young people [27, 28].

One of the targets of the UN Sustainable Development Goals (SDG) is to reduce the global maternal mortality ratio to fewer than 70 per 100,000 live births by 2030. Reaching this target in Nigeria would require a maternal mortality ratio reduction of 7.5% per year [29]. The mortality burden among 15–24 year-old women due to unsafe abortion, along with the limitations in the previous studies, underscores the importance of understanding what factors influence pregnancy termination amongst this age group in Nigeria. Such knowledge would be of value to inform the development of tailored interventions and programmes to reduce the burden of maternal morbidity and mortality [30] particularly in Northern Nigeria- the region with the highest maternal and under-five mortality in the country [31]. Our aim was to examine the trends in prevalence of pregnancy termination, and the individual and contextual-level factors that influence pregnancy termination among 15–24 year-old women in Nigeria.

## Methods

### Study design

Cross-sectional study design.

### Data source and sample of participants

Data for this study were based on four datasets from the 2003, 2008, 2013 and 2018 Nigerian Demographic Health Survey (NDHS). The NDHS is a nationally-representative household survey implemented by the Nigeria National Population Commission and Measure DHS with financial and technical support from global development agencies such as USAID [32]. The NDHS data were collected using a 2-phase multistage, stratified and clustered sampled design based on a list of enumerated area (EAs). At the first stage, a random sample of clusters were drawn from the 36 states and Federal Capital Territory (FCT) using the census files. In the second stage, a random sample of households was selected from each cluster using a household list from a national master sampling frame. Details of the NDHS sampling procedures and questionnaire are available elsewhere [32]. The data used in the analyses were weighted in order to adjust for differences in the probability of selection and to adjust for non-response. A total of 121,774 women aged 15–49 were interviewed in the four surveys. The NDHS includes all women age 15–49 in the sample households, and therefore can include minors aged 15–16 years. The sample used in this analysis was limited to women aged 15–24 years giving a total sample size of 45,793. Sample characteristics are given in annex (i).

### Outcome variable

The outcome variable for this study was ‘ever termination of pregnancy’ reported by the respondent. Pregnancy termination is defined in the NDHS as any pregnancy that resulted in a miscarriage, abortion or stillbirth [32]. This definition is commonly used in studies in SSA in order to mitigate social desirability bias [13, 20, 33]. Young women may recast their experience of induced abortion to a miscarriage or stillbirth as it is less stigmatised and not prohibited by law.

### Explanatory variables

The explanatory variables (both individual and contextual) were selected on the basis of an association with the outcome reported in studies identified in a scoping review of the literature to inform the analysis, and on the availability of variables in the NDHS datasets (Table 1). The community-level factors (community-wealth index, community-level education, community-level mass media (radio, TV and newspaper) exposure and community-level contraceptive prevalence) and state-level factors (state-level wealth index, state-level education and state-level mass media exposure) among 15–24 year-old women were derived from individual-level measures. For example, contraceptive prevalence among 15–24 year-old women at community level was derived from the individual-level variable ‘contraceptive use’ and used as a proxy for reproductive health service access among 15–24 year-old women within a community/cluster.

**Table 1:** Description of explanatory variables

Variables	Definitions
Year of survey	Survey years classified into 4 groups 2003; 2008, 2013 and 2018.
Age group	Age group of respondents at the time of the survey classified into 2 groups: 15–19 and 20–24 years
Education	Education attainment of the women classified into 3 groups: no education, primary; secondary and above
Marital status	Classified into 4 groups: never married; currently married; living with a partner (but not legally married) and previously married (widowed, divorced, separated).
Wealth index	Constructed by the DHS using principal components analysis based on household assets. Classified into five groups: Poorest; Poorer; Middle; Richer and Richest.
Contraceptive use	Denotes percentage of women in a cluster/state who currently use contraceptive methods. At individual level, this is a dichotomous variable classified as users and nonusers
Age at first sex	Classified into 2: Never had sex; Less than age 15; 15–17 and 18+ years
Forced sex	Ever forced to perform unwanted sexual acts. Classified into 2 groups: Yes and No
Frequency of reading newspaper	Classified into 3 groups: Not at all; Less than once a week and at least once a week or more.
Frequency of listening to radio	Classified into 3 groups: Not at all; Less than once a week and at least once a week or more.
Frequency of watching TV	Classified into 3 groups: Not at all; Less than once a week and at least once a week or more.
No of living children	Number of living children at the time of the interview. Classified into 4 groups; 0, 1, 2 and 3^+^
Ethnicity	Ethnic origin of respondents classified into 4 groups: Hausa; Igbo; Yoruba and Others
Religion	Religious affiliation of respondents, classified into 3 groups: Catholic; other Christians and Islam.
Place of Residence	Place of residence, grouped into 2: Urban and rural
Region	Region of residence grouped into 6: North Central, North East; North West; South East; South South; South West.

### Data analysis

Trends in pregnancy termination across the four datasets (2003–2018) and associations between each of the explanatory variables with pregnancy termination were examined using bivariate analysis. All bivariate associations were based on weighted percentages to enable examination of patterns across different regions of the country. Individual and contextual predictors of pregnancy termination were examined using a three-level binary logistic regression analysis on pooled data for all four datasets and are reported as adjusted odds ratios (aOR) with 95% confidence intervals (CI). We assessed potential collinearity between the explanatory variables, before adding variables to the multilevel models. None of the variables included in the multivariate model were highly correlated, and there was no evidence of multicollinearity.

The multilevel analysis placed particular emphasis on community and state variations in pregnancy termination among young people in Nigeria. The pooled NDHS data used in the multilevel analysis have a hierarchical structure with individuals nested within communities (clusters) which in turn were nested within states. In the multilevel analysis applied in this paper, states constituted the highest (third) level (*n* = 36), while communities (i.e clusters) within states constituted the second level. The general form of the three-level random intercepts [34] logistic regression model used may be expressed as:

1 $$ Logit\kern0.5em {\pi}_{ijk}=X{'}_{ijk}\beta +{u}_{jk}+{v}_k $$

where: *π*_ijk_ is the probability of pregnancy termination (ever had terminated pregnancy) for an individual *i*, in the *j*^*th*^ community in the *k*^*th*^ state; *X’*_*ijk*_ is the vector of covariates which may be defined at the individual, community or state level; β is the associated vector of usual regression parameter estimates; and the quantities *v*_*k*_*,*and *u*_*jk*_ are the residuals at the state and community level, respectively. These are assumed to have normal distribution with mean zero and variances *σ*^*2*^_*v*_ and *σ*^*2*^_*u*_ [34].

The estimates of state and community level variances are used to calculate intra-unit correlation coefficients (ICC) to examine the extent to which pregnancy termination among 15–24 year-olds are clustered within states (or communities within states) in Nigeria. We note that the intra-community correlation includes state correlations since individuals within the same community are also within the same state [35]. Therefore, the formulae for intra-community (*ρ*_*u*_) and intra-state (*ρ*_*v*_) correlation coefficients are given by:

2 $$ {\rho}_u=\frac{\sigma_u^2+{\sigma}_v^2}{\sigma_v^2+{\sigma}_u^2+{\sigma}_e^2} $$

3 $$ {\rho}_v=\frac{\sigma_v^2}{\sigma_v^2+{\sigma}_u^2+{\sigma}_e^2} $$

where:

σ_v_^2^ - is the state level variance;

σ_u_^2^ - is the community (cluster) level variance; and

σ_e_^2^ - is the individual level variance.

The level-1 residuals (for a logistic regression model), e_ijk_, are assumed to have a standard logistic distribution with a mean of zero and variance of π^2^/3, where π is the constant 3.1416 (See [35]). The multilevel analysis was carried out using MLwiN software and estimates of parameters based on second order Predictive Quasi-Likelihood (PQL) procedure [36].

## Results

### Trends in pregnancy termination among 15–24 year-old women in Nigeria 2003–2018

**Fig. 1:**
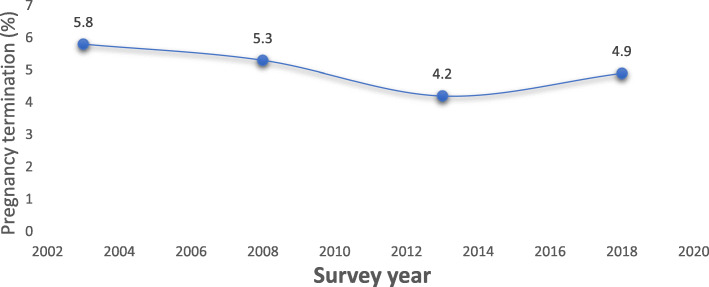
Pregnancy termination among young women aged 15–24 years 2003–2018 in Nigeria

Overall, there was significant differences in pregnancy termination between the survey years. Pregnancy termination among 15–24 year olds significantly declined (*P* < 0.001) from 5.8% in 2003 to 4.2% in 2013 then increased up to 4.9% in 2018 in Nigeria (Fig. 1). The overall declining trend was apparent for most explanatory variables examined in the bivariate analysis. The results in Table 2 show a differential pattern in pregnancy termination such that young women who had no education or primary education only, those from the poorer/poorest households, those who had sexual debut at age younger than 15 years, women from Hausa ethnic group, women of Islamic faith and those who live in rural areas showed no substantial decline in pregnancy termination and in some cases increased across the survey years, contrary to patterns observed among their counterparts. For example, pregnancy termination decreased from 5.7% in 2003 to 3.5% in 2018, among urban women, but increased from 5.8% in 2003 to 6.0% in 2018 among their rural counterparts. Similar pattern exists across the ethnic groups such that pregnancy termination increased from 6.6% in 2003 to 7.0% in 2018 among women of Hausa ethnic background, but decreased from 4.0% in 2003 to 1.7% in 2018 among women from the Igbo ethnic group (Table 2).

**Table 2:** Pregnancy termination among young women aged 15–24 in Nigeria, 2003–2018 NDHS

Variables	2003	2008	2013	2018	Total	
	Weighted %	Weighted %	Weighted %	Weighted %	Weighted%	Unweighted cases
Age group	**	**	**	**		**
15–19	3.4	2.3	2.0	2.2	2.2	24,627
20–24	8.6	8.5	6.6	8.3	7.8	21,105
Education	*	**	**	**		**
No Education	7.7	5.9	5.4	7.8	6.5	12,979
Primary	6.5	7.0	6.7	7.2	6.9	6080
Secondary and above	4.3	4.5	3.1	3.2	3.5	26,673
Marital status	**	**	**	**		**
Never married	2.3	2.6	1.3	0.9	1.5	25,626
Currently married	9.9	8.2	7.5	10.2	8.7	18,546
Living with a partner	11.9	15.5	11.1	12.9	12.5	853
Previously married	4.3	6.1	7.3	9.5	7.6	706
Wealth index	ns	ns	*	**		**
Poorest	8.0	5.5	4.6	6.5	5.8	8165
Poorer	5.9	5.7	5.1	6.1	5.7	9186
Middle	5.1	4.8	4.2	6.0	5.1	9878
Richer	5.7	5.9	4.0	3.7	4.5	9998
Richest	4.8	4.4	3.4	2.4	3.4	8505
Ever use of any method of contraceptive	**	**	**	**	*	**
Never used	4.3	4.0	1.4	4.7	4.3	27,254
Used	12.0	12.4	4.6	7.1	9.9	3880
Age at first sex	**	**	**	**		**
Never had sex	0.0	0.0	0.0	0.0	0.0	17,359
Less than 15	9.7	8.6	7.4	11.1	8.9	6791
15–17	9.2	8.7	6.7	8.5	8.0	13,355
18+	6.2	6.8	5.6	6.5	6.2	8148
Forced sex	–	ns	*	ns		**
No	0.0	5.7	4.8	6.0	5.3	19,766
Yes	0.0	12.0	9.5	11.3	10.7	1248
Frequency of reading newspaper	ns	ns	**	**		**
Not at all	6.2	5.2	4.8	5.3	5.2	35,696
Less than once a week	4.0	6.6	2.5	3.1	3.7	5663
At least once a week or more	5.5	4.2	2.5	2.8	3.5	4160
Frequency of ** listening to radio	ns	*	*	ns		*
Not at all	6.1	5.4	4.2	5.0	4.9	17,607
Less than once a week	5.8	6.7	5.0	5.0	5.3	10,209
At least once a week or more	5.7	4.8	3.6	4.6	4.5	17,819
Frequency of *** watching TV	ns	ns	**	**		**
Not at all	6.1	5.5	4.7	6.3	5.6	21,279
Less than once a week	3.2	5.9	4.3	3.9	4.4	7864
At least once a week or more	6.1	4.9	3.7	3.5	4.1	16,483
No of living *** Children	**	**	**	**		**
0	3.9	3.6	2.7	2.4	2.9	29,625
1	8.8	8.3	7.5	9.8	8.6	8693
2	8.5	8.8	5.7	10.4	8.3	4863
3+	12.1	7.5	6.7	7.6	7.6	2551
Ethnicity	*	**	**	**		**
Hausa	6.6	6.3	5.7	7.0	6.4	11,058
Igbo	4.0	3.1	2.4	1.7	2.5	6499
Yoruba	2.0	1.8	2.8	2.4	2.4	5895
Others	6.7	6.4	4.4	5.1	5.4	22,272
Religion	*	ns	**	**		**
Catholic	5.5	4.5	2.6	3.2	3.6	5179
Other Christians	4.9	5.9	3.8	3.3	4.3	18,222
Islam	6.6	5.1	4.8	6.1	5.5	22,207
Place of Residence	ns	*	**	**		**
Urban	5.7	4.6	3.4	3.5	3.8	17,109
Rural	5.8	5.6	4.8	6.0	5.5	28,623
Region	**	**	**	**		**
North Central	4.5	3.6	1.8	4.5	3.7	8356
North East	9.2	6.0	4.8	6.0	5.9	8503
North West	6.4	5.6	5.7	6.7	6.1	10,910
South East	2.1	2.8	2.5	1.3	2.2	5401
South South	7.2	12.4	4.7	4.6	6.8	6515
South West	2.7	2.2	2.9	2.7	2.7	6047
Total						**45,732**

* significant at 5% level (*p* < 0.05); ** = *p* < 0.001; ns = Non-significant

### Percentage distribution of pregnancy termination in women aged 15–24 years in Nigeria by socio-demographic and potential explanatory factors

The percentages of women aged 15–24 years who reported ever termination of pregnancy for all years combined are shown in Table 2. For all years combined, it shows that a higher percentage of women with no education (6.5%) and primary education (6.9%) reported termination of pregnancy compared with young women with secondary education and above (3.5%). Women of Hausa ethnic group (6.4%) were more likely to report pregnancy termination than those from Igbo (2.5%) and Yoruba (2.4%) ethnic groups. A higher proportion of women of Islamic faith (5.5%) reported pregnancy termination than the Catholic (3.6%) and other Christians (4.3%). More women who reported ‘ever use of contraceptives’ (9.9%) reported pregnancy termination than women who reported ‘never used contraceptives’ (4.3%). Regional differences in pregnancy termination across the survey years are apparent such that young women from the Northern region were more likely to report terminated pregnancy than those from the Southern region.

### Multilevel results of the predictors of pregnancy termination in women aged 15–24 years in Nigeria

Predictors of pregnancy termination based on a three-level multilevel model, taking State as Level 3 and Cluster as Level-2 are presented in Table 3. Only significant factors, after controlling for the effect of other covariates, were included in the final model. Some individual-level factors, such as religion, ceased to be significant once other factors were included in the model. Both wealth and region ceased to be significant, once a significant contextual factor – i.e. contraceptive prevalence among young women in a cluster – was included in the model. The interaction of individual variables with year of survey were tested to assess whether the effects of any of the predictors varied across time, but none were significant. Of all contextual factors considered, including wealth, education, contraceptive prevalence (proxy for RH service access by young women), media exposure at both cluster and state levels, only contraceptive prevalence at cluster level was significant.

**Table 3:** Multilevel parameter estimates and average odds ratios for pregnancy termination

PARAMETER	ESTIMATE	S. E	AOR	95% CI of AOR		
Const	−4.57	0.186				
***Year (2003)***						
2008	−0.13	0.105	0.88	[0.71,	1.08]	
2013	−0.41	0.188	0.66	[0.46,	0.96]	*
2018	0.16	0.103	1.17	[0.96,	1.44]	
***Age group (15–19)***						
20–24	0.85	0.076	2.34	[2.02,	2.72]	*
***Education (none)***						
Primary	0.35	0.089	1.42	[1.19,	1.69]	*
Secondary	0.12	0.094	1.13	[0.94,	1.36]	
***Marital status (never married)***						
Currently married	1.10	0.102	3.00	[2.46,	3.67]	*
Living with a partner	1.14	0.162	3.13	[2.28,	4.30]	*
Previously married	0.64	0.206	1.90	[1.27,	2.84]	*
***Watch TV (Never)***						
less than once a week	0.11	0.095	1.12	[0.93,	1.34]	
at least once a week	0.31	0.081	1.36	[1.16,	1.60]	*
***Ethnicity (Hausa)***						
Igbo	−0.40	0.165	0.67	[0.49,	0.93]	*
Yoruba	−0.66	0.172	0.52	[0.37,	0.72]	*
Others	−0.08	0.089	0.92	[0.78,	1.10]	
***Number of children (none)***						
One	−0.25	0.077	0.78	[0.67,	0.91]	*
Two	−0.50	0.094	0.61	[0.50,	0.73]	*
3 and more	−0.84	0.121	0.43	[0.34,	0.55]	*
***Age at first sex (18+*)**						
Never had sex	−5.55	1.060	0.00	[0.00,	0.03]	*
Less than 15	0.84	0.094	2.32	[1.93,	2.78]	*
15–17	0.50	0.790	1.65	[0.35,	7.76]	
***Contextual factors***						
contracep prev_cluster	1.43	0.231	4.18	[2.66,	6.57]	*
***Random effects***						
Level 3 - State	0.10*	0.033	(ICC = 0.03)			
Level 2 - Cluster	0.22*	0.056	(ICC = 0.09)			

* - significant at 5% level (*p* < 0.05)

The multilevel results shown in Table 3 confirm the findings from the crude analysis of trends over time and show a declining trend in pregnancy termination among women aged 15–24 years up to 2013, which reverses in 2018, after significant individual and contextual predictors were controlled for. There was a 34% reduction in the odds of pregnancy termination in 2013 compared with 2003, but no evidence of a significant difference in 2008 and 2018 compared with 2003.

Living in communities with higher contraceptive prevalence increased the odds of termination compared with communities with lower contraceptive prevalence (aOR = 4.2; 95% CI 2.7–6.6).

The odds of pregnancy termination were 2.34 higher among women aged 20–24 than women aged 15–19. Other strong predictors were marital status, sexual debut before age 15 years and contraceptive prevalence. The odds of pregnancy termination were triple for women who were currently married or living together, and almost double for women who were previously married but not living together compared with young women who have never married. Young women who had initiated sexual activity before age 15 had 2.3 times higher odds of pregnancy termination than those who initiated sexual activity at a later age of 18 years or older. Young women living in communities (i.e. clusters) with higher contraceptive prevalence among young women (a proxy for higher access of reproductive health services by young women) had significantly higher odds of pregnancy termination than those in communities with lower contraceptive prevalence.

The odds of pregnancy termination were 1.4 times higher for women with primary education compared with young women with no education, but there was no evidence of a significant difference between women with secondary education and women with no education. Women from Igbo and Yoruba ethnic groups had significantly lower odds of pregnancy termination than women of Hausa ethnicity. There was evidence of a decline in the odds of pregnancy termination as the number of living children increased. There was some evidence that more frequent exposure to TV was associated with higher odds of pregnancy termination.

### Variations in pregnancy termination among young women across communities and states in Nigeria

There were significant variations in pregnancy termination across communities (i.e clusters) and states in Nigeria. The cluster-level random variances from the variance components model (before controlling for the effect of any individual or contextual predictors of pregnancy termination) suggest that about 17% (i.e (0.417 + 0.263)/(0.417 + 0.263 + 3.29)) of the total variation in pregnancy termination among the young women in Nigeria is attributable to community (i.e. cluster) level factors, while about 7% (i.e (0.263)/(0.417 + 0.263 + 3.29) is attributable to state level factors, with the remaining 76% attributable to individual-level factors.

**Fig. 2:**
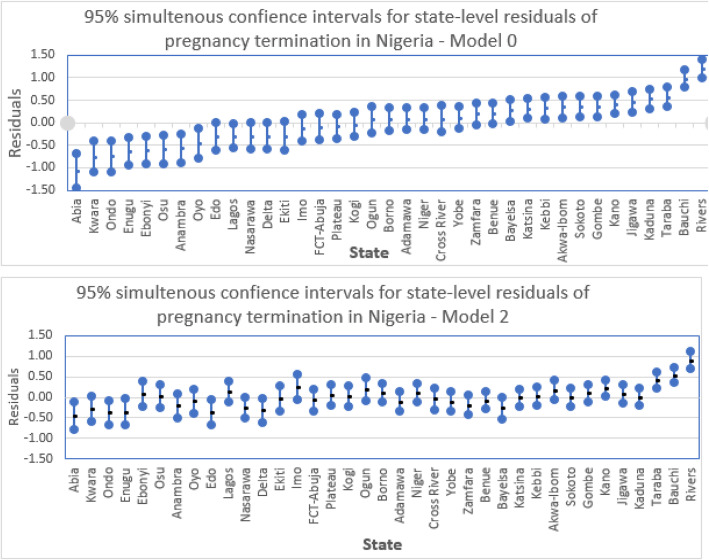
State variations in pregnancy termination on pooled data for 2003–2018

After controlling for significant observable individual and contextual factors, about 9% (i.e. (0.22 + 0.10)/(0.22 + 0.10 + 3.29) and 3% (i.e (0.10)/(0.22 + 0.10 + 3.29)) of the total unexplained variation is attributable to unobserved cluster and state-level factors, respectively, with the remaining 88% attributable to unobserved individual characteristics. The state variations in pregnancy termination are highlighted in Fig. 2 showing 95% simultaneous confidence intervals of state residuals [37]. Non-overlap of confidence intervals suggests a significant difference in odds of pregnancy termination between the states. The states are ordered from lowest to highest risk of pregnancy termination before any of the predictors are controlled for (Model 1). Before controlling for any predictors, states with lowest risk of pregnancy termination are predominantly in the South West, South East and North Central regions, while those with highest risk are predominantly in North West, North East and South South. Although, noticeably reduced, significant state-level variations remain when significant predictors are controlled for (Model 2).

## Discussion

To our knowledge, this is the first comprehensive analysis of the NDHS from 2003 through 2018 that focuses specifically on the influence of both individual and contextual-level factors on pregnancy termination among women aged 15 to 24 years, a demographic group that is often overlooked in terms of research and public health interventions. Our key findings reveal a declining trend in pregnancy termination between 2003 and 2013, with a reversal in trend in 2018. Additionally, whilst the overall trend in pregnancy termination between 2003 and 2018 showed a decline, amongst some groups of young women there were disparities in the trends particularly for measures of socioeconomic status: young women with no education/primary education, and those from the poorest/poorer households showed no decline in pregnancy termination as opposed to those who were educated or were from wealthier households. The observed trend in pregnancy termination mirrored the trends in contraceptive use, and age at sexual debut, which were found to be significant factors associated with pregnancy termination in the multivariable model. The declining trend in pregnancy termination persisted especially from 2003 to 2013 after individual and contextual factors were controlled for. One potential explanation for these observed declining trends in termination could be due to the impact of the nationwide introduction of the school-based Family Life and HIV Education (FLHE) curriculum in 2003, which was designed amongst other things to provide young people with sexual health literacy skills. Recent evidence shows that in states where the FLHE was effectively implemented, an improvement in sexual health knowledge of youth was found [38]. However, overall, implementation of this programme has dwindled in recent years due to limited continued financial and technical support from government agencies [38]. Moreover, in some regions the FLHE has been fraught with setbacks as individual states can modify the content of the curriculum coupled with the minimal acceptance of sexuality education particularly in regions with highly conservative religious and cultural values [39, 40]. The sub-optimal implementation may be a factor contributing to the increase in early sexual initiation and pregnancy termination we observed in the data from 2013 to 2018 [32]. The declining trend between 2003 and 2013 we found, albeit with lower percentages, is similar to another study using DHS data for Nigeria but limited to married women of a wider age range (15–49 years) [13].

Surprisingly, we found a significant decline in contraceptive use from 2003 to 2018 for 15–24 year-old women. This important finding has not been evident in previous studies that aggregated data for a wider age range of women (15–49 years) in Nigeria [32], perhaps due to an ecological fallacy [13, 14, 16, 17]. This calls into question whether the current National Reproductive Health Policy and Strategy to achieve Quality Reproductive Health and Sexual Health for All Nigerians including the provision of free family planning services in public facilities [41] has benefited all women of reproductive age in Nigeria. Our findings indicate that 15–24 year-old women are still being left behind on reproductive health matters despite increasing global attention to prioritising their health.

Another interesting finding was the influence of contextual level factors on pregnancy termination. Although the majority of the variation was at the individual level, significant variation in pregnancy termination was found between women aged 15–24 years across communities and states. While some community and state-level variables were not significantly associated with pregnancy termination, contraceptive prevalence at community level, which we derived from the individual-level variable ‘contraceptive use’ and then used as a proxy for reproductive health service accessibility within a community, was strongly positively associated with pregnancy termination. This suggests that communities with higher levels of reproductive health services, indicated by higher prevalence of contraceptive use, are linked to higher prevalence of pregnancy termination. This may indicate that despite availability of reproductive health services and contraceptives, factors such as pressure to have children and stigma surrounding non-marital sexual activity may hinder accessing them [42]. Interventions to discourage early marriage and dispel misconceptions and stigmatising attitudes towards contraceptive use such as scientifically accurate Comprehensive Sexuality Education (CSE) could help overcome these barriers and should be encouraged [18, 42]. However, these results contradict the findings of a previous study in Nigeria which found that residing in a community with contraceptive use at levels above the median for the community was associated with a lower likelihood of terminating pregnancy [20]. The disparity in the findings could again be attributable to the fact that the previous study [20] focused on a wider age range of women (15–49 years). The integration of a wider age range of women in the study may have influenced the results as evidence has shown that older women have more contraceptive utilisation rate compared to younger women [12, 43].

Furthermore, at the individual level, we found that pregnancy termination was higher among women aged 20–24 than those aged 15–19 after controlling for confounding factors. This is not surprising as the NDHS report in 2018 revealed that the median age at first sexual intercourse is 17.2 years for women of reproductive age in Nigeria [32]; meaning that older women are more likely to be sexually active or exposed than their younger counterparts, which predisposes them to unwanted pregnancy, leading to abortions in many cases [44, 45]. The higher risk of pregnancy termination among older women persisted, even after the age of sexual debut was controlled for. It is possible that younger women are less likely to report or seek abortion services due to cultural, socio-economic and institutional barriers [42, 44]. Several other studies have reported similar findings [13, 15, 33].

Marital status was found to be a strong predictor of pregnancy termination. Currently married women aged 15–24 years, those living with a partner or previously married and not living with a partner were more likely to terminate pregnancy than never-married women. This could be linked to social desirability bias as never-married 15–24 year old women may be reluctant to report pregnancy termination due to social stigma surrounding non-marital sexual activity or use of contraceptive [46]. However, this is inconsistent with the findings of previous school-based studies in Nigeria [16, 18] and facility-based studies among older women in Ethiopia [23], Burkina Faso [47] and Ghana [22], which found that unmarried youth are more likely to terminate a pregnancy than their married counterparts. The conflicting findings could be due to the difference how pregnancy termination was defined. In this study it was defined as any pregnancy that resulted in a miscarriage, abortion or stillbirth, which may not accurately reflect the main outcome of interest (induced abortion) [48]. While the school-based and facility-based studies measured only abortion. Although evidence from the DHS methodological report shows that only 2–5% of women who reported their pregnancy ending in miscarriage during a face-to-face interview changed their responses to induced abortion in a self-administered questionnaire [49].

Also, the results of this study revealed that 15–24 year-old women from both Igbo and Yoruba ethnic groups (predominantly in the South) have a lower tendency for pregnancy termination than those from Hausa ethnic group (predominantly in the North). The states with the highest risk of pregnancy termination are predominantly in the North rather than the South. This could conceivably be attributable to the disparity in the level of education, rates of early marriage, and cultural norms/beliefs across different ethnic groups in Nigeria [50]. For example, the average age at marriage for most girls in Northern Nigeria is 15 years compared with a median age of 24 years in the Southern regions of the country, Girls in the Southern regions are more likely to access formal education, while in the Northern regions, girls are less likely to access formal education and, consequently, experience sexual initiation at earlier ages [51–53]. It is also worth stressing that maternal mortality and under-five mortality rates in Nigeria vary considerably with some Northern states reporting nearly 50% more deaths for both indicators than the national average [31, 54]. Furthermore, the North-South disparity in education supports our findings that young women educated to primary level and above are less likely to terminate a pregnancy than their uneducated counterparts. Young women who are educated are more likely to have access to information and available services and possibly, delay childbearing and marry later than those with no education. These findings are consistent with the findings of other studies [8, 15, 22, 24]. Also, in Ethiopia, evidence has shown that women with higher education were less likely to have induced abortion [55]. Lack of education and early sexual initiation/exposure, without doubt, expose young women to unintended pregnancy; leading to pregnancy termination [16].

### Strengths and limitations

This study has key strengths due to utilising nationally representative datasets (from 2003 to 2018), a large sample size and the analysis adjusted for both individual and contextual (community and state) level factors. The analysis of variance indicated that around 24% of the variation in pregnancy termination among young women was due to the contextual level factors; supporting the value of including these in multivariable analyses. However, it, is not void of limitations that could have potential implications for the findings. One of the shortcomings is due to the cross-sectional study design which does not enable us to infer causal relationships between predictors and the outcome variable [13, 56]. Another is that the NDHS depends on self-reported data collection, which can be subject to bias especially in a setting like Nigeria where abortion is legally restrictive; making pregnancy termination more likely to be under-reported. This could be averted by the use of indirect questioning techniques in elucidating responses to the question on pregnancy termination [20]. Furthermore, a terminated pregnancy could mean any type of pregnancy outcome other than one resulting in a live birth including: stillbirths, miscarriages and abortions, which could lead to overestimation of terminated pregnancy. Finally, this study did not control for facility-level factors which previous studies have suggested influence pregnancy termination [25, 57]. However, our analysis indicated that residual confounding due to unobserved contextual level factors is only 12%, therefore, the impact of this may be small.

## Conclusions

This study provides insight into the trends and individual/contextual level predictors of pregnancy termination among women aged 15–24 years in Nigeria. Although most predictors of pregnancy termination occurred at the individual level, community and state level factors also predisposed women aged 15–24 years to terminate pregnancy. While there was a general decline in pregnancy termination among women aged 15–24 years in Nigeria, those with lower socioeconomic status may not have benefited from the current policy and programmes to the same extent as women of higher status. Consideration of whether a shift in policy direction and service provision is required, and these findings also highlights the need for further research to understand why the available services are not benefitting all women of reproductive age. The study suggests the need to shift from the current one size fits all service provision to targeted and tailored services to meet the SRH health needs of young women. Integrating an innovative community-based health programme such as the Health Extension Program (HEP) into the existing services to expand access to SRH services may be a viable option. The findings show the need for multifaceted programs to address the influence of socio-cultural norms and contextual factors that influence pregnancy termination among young women in Nigeria. Policy interventions such as scientifically accurate Comprehensive Sexuality Education to delay early sexual debut and build agency to use contraception among young women especially those with no education, from the Hausa ethnic group and who reside in rural communities should be encouraged. This could reduce poor maternal health outcomes especially in Northern Nigeria with highly conservative religious and cultural values. Together these could make a positive impact on the SDG target to reduce maternal mortality.

## Supplementary information

**Additional file 1.** Background sample distribution of young people aged 15–24 by selected socio-demographic factors Nigeria, 2003 to 2018 NDHS.

## Data Availability

The datasets analysed during the current study are available from the corresponding author on reasonable request.

## Abbreviations

aOR: Adjusted Odds Ratio
CSE: Comprehensive Sexuality Education
EAs: Enumerated areas
FLHE: Family Life and HIV Education
FCT: Federal Capital Territory
HEP: Health Extension Program
ICC: Intraclass Correlation Coefficient
CI: Confidence interval
NHREC: National Health Research Ethics Committee of Nigeria
NPC: National Population Commission
NDHS: Nigerian Demographic and Health Surveys
PQL: Predictive (or penalized) Quasi-Likelihood.
QR-GCRF: Global Challenges Research Fund
RH: Reproductive Health
SRH: Sexual and Reproductive Health
SSA: Sub-Saharan Africa
STI: Sexually Transmitted Infection
SDG: Sustainable Development Goals
USAID: United States Agency for International Development
WHO: World Health Organization
