# Supplementary material for: Trends in and predictors of pregnancy termination among 15–24 year-old women in Nigeria: a multi-level analysis of demographic and health surveys 2003–2018
Source: BMC Pregnancy Childbirth. 2020 Sep 22;20:550. doi: 10.1186/s12884-020-03164-8 (PMC7507716; doi:10.1186/s12884-020-03164-8)
Supplement: Supplementary file 1 — Additional file 1. Background sample distribution of young people aged 15–24 by selected socio-demographic factors Nigeria, 2003 to 2018 NDHS. [file 12884_2020_3164_MOESM1_ESM.docx]

**Additional file 1: Background sample distribution of young people aged 15-24 by selected** **socio-demographic factors Nigeria, 2003-2013 NDHS.**

| Variables | Weighted  Percentages (%) | Unweighted  cases |
| --- | --- | --- |
| Year of survey |  |  |
| **2003** | 7.0 | 3213 |
| **2008** | 26.2 | 12635 |
| **2013** | 33.3 | 14678 |
| **2018** | 33.4 | 15267 |
| Age |  |  |
| **15-19** | 53.6 | 24668 |
| **20-24** | 46.4 | 21125 |
| Education |  |  |
| **No Education** | 29.0 | 13003 |
| **Primary** | 12.9 | 6089 |
| **Secondary and above** | 58.1 | 26703 |
| Marital status |  |  |
| **Never married** | 55.0 | 26655 |
| **Married** | 41.8 | 18578 |
| **Living together** | 1.7 | 853 |
| **Not living together** | 1.5 | 706 |
| Wealth index |  |  |
| **Poorest** | 17 | 8178 |
| **Poorer** | 19.5 | 9202 |
| **Middle** | 20.5 | 9883 |
| **Richer** | 22.0 | 10011 |
| **Richest** | 21.0 | 8519 |
| Ethnicity |  |  |
| **Hausa** | 27.6 | 11081 |
| **Igbo** | 14.3 | 6508 |
| **Yoruba** | 13.9 | 5900 |
| **Others** | 44.2 | 22296 |
| Religion |  |  |
| **Catholic** | 11.6 | 5185 |
| **Other Christians** | 37.1 | 18240 |
| **Islam** | 51.3 | 22243 |
| Place of Residence |  |  |
| **Urban** | 40.4 | 17135 |
| **Rural** | 59.6 | 28658 |
| Region |  |  |
| **North Central** | 14.8 | 8364 |
| **North East** | 15.6 | 8514 |
| **North West** | 28.7 | 10934 |
| **South East** | 11.5 | 5406 |
| **South South** | 13.9 | 6522 |
| **South West** | 15.6 | 6053 |
| Total |  | **45793** |
